# Supplementary material for: Acoziborole resistance associated mutations in Trypanosoma brucei CPSF3
Source: PLoS Pathog. 2026 Mar 3;22(3):e1013764. doi: 10.1371/journal.ppat.1013764 (PMC12970967; doi:10.1371/journal.ppat.1013764)
Supplement: S1 Fig — Other sites assessed by oligo targeting are shown in red text. Sites associated with benzoxaborole resistance in Apicomplexan parasites (Plasmodium falciparum, PF3D7_1438500, Y408S and D470N; Toxoplasma gondii, TGME49_285200, Y328H, Y483N, S519C and E545K) and noted in the main text are shown in light blue text. The Thermus thermophilus protein used as template for homology modelling (BAD70075) and the human CPSF3 (AAF00224.1) are also shown for reference. (PDF) [file ppat.1013764.s001.pdf]

```
T. brucei       ILIAESTNGIRELESREERESLFTTWVHDVVKGGGRCLVPVFALGRAQELLLILEEYWEAHKELQHIPIYYASSLAQRCM 304
P. falciparum   VLICEGTYGIKVHDDRKKREIRFLNILTSMINNKGKVLLPVFALGRAQELLLILEEHWDKNKHLQNIPIFYISSMATKSL 324
T. gondii       LLICESTYGIHVHDDRQLRERRFLKAVVDIVNRGGKCLLPVFALGRAQELLLILEEYWTAHPEIRHVPILFLSPLSSKCA 400
T. thermophila  LVLAEGTYGDRPHRPYRETVREFLEILEKTLSQGGKVLIPTFAVERAQEILYVLY---THGHRLPRAPIYLDSPMAGRVL 258
human           ILIIESTYGTHIHEKREEREARFCNTVHDIVNRGGRGLIPVFALGRAQELLLILDEYWQNHPELHDIPIYYASSLAKKCM 279

T. brucei       KLYQTFVSAMNDRVKKQHENHRNPFVFKYIQSLLDTRS-----FEDTGPCVVLASPGMLQSGISLELFERWCGDKRNGII 379
P. falciparum   CIYETFINLCGEFVKKVVNEGKNPFNFKYVKYAKSLESISSYLYQDNNPCVIMASPGMLQNGISKNIFNIIASDKKSGVI 404
T. gondii       VVFDAFVDMCGEAVRSRALRGENPFAFRFVKNVKSVEAARVYIHHD-GPAVVMAAPGMLQSGASREIFEAWAPDAKNGVI 479
T. thermophila  SLYPRLVRYFSEEVQAHFLQGKNPFRPAGLEVVEHTEA-SKALNRAPGPMVVLAGSGMLAGGRILHHLKHGLSDPRNALV 337
human           AVYQTYVNAMNDKIRKQI-NINNPFVFKHISNLKSMDH-----FDDIGPSVVMASPGMMQSGLSRELFESWCTDKRNGVI 353

T. brucei       VAGYCVDGTIAKDILSKPREITKPDGKVLPLRMRTIQSVSFSAHSDGRQTRDFIQALPKTKHVILVHGNVGAMGQL 455
P. falciparum   LTGYTVKGTLADELKTEPEFVTIND-KVVKRKCR-FEQISFSAHSDFNQTKTFIEKLK-CPNVVLVHGDKNELNRL 477
T. gondii       LTGYSVKGTLADELKREPETIQLPD-RVLRRRCS-FEMISFSAHSDYQQTQEFIGKLK-VPNVVLVHGERGEMRRL 552
T. thermophila  FVGYQPQGGLGAEIIARPPAVRILG-EEVPLRASVHTLGGFSGHAGQDE---LLDWLQGEPRVVLVHGEEEKLLAL 409
human           IAGYCVEGTLAKHIMSEPEEITTMSGQKLPLKMS-VDYISFSAHTDYQQTSEFIRALK-PPHVILVHGEQNEMARL 427
```

Supplementary Fig. 1: The protein sequence alignment shows the region of *T. brucei* CPSF3 (Tb927.4.1340) that includes the mutations in the acoziborole resistant triple-mutant (green background). Other sites assessed by oligo targeting are shown in red text. Sites associated with benzoxaborole resistance in Apicomplexan parasites (*Plasmodium falciparum*, PF3D7_1438500, Y^{408}S and D^{470}N; Toxoplasma gondii, TGME49_285200, Y^{328}H, Y^{483}N, S^{519}C and E^{545}K), and noted in the main text are shown in light blue text. The *Thermus thermophilus* protein used as template or homology modelling (BAD70075) and the human CPSF3 (AAF00224.1) are also shown for reference.
