# Supplementary material for: Acoziborole resistance associated mutations in Trypanosoma brucei CPSF3
Source: PLoS Pathog. 2026 Mar 3;22(3):e1013764. doi: 10.1371/journal.ppat.1013764 (PMC12970967; doi:10.1371/journal.ppat.1013764)
Supplement: S2 Fig — Pose 2, for the 3IEM-based model has the greatest impact following mutations at these sites. (PDF) [file ppat.1013764.s002.pdf]

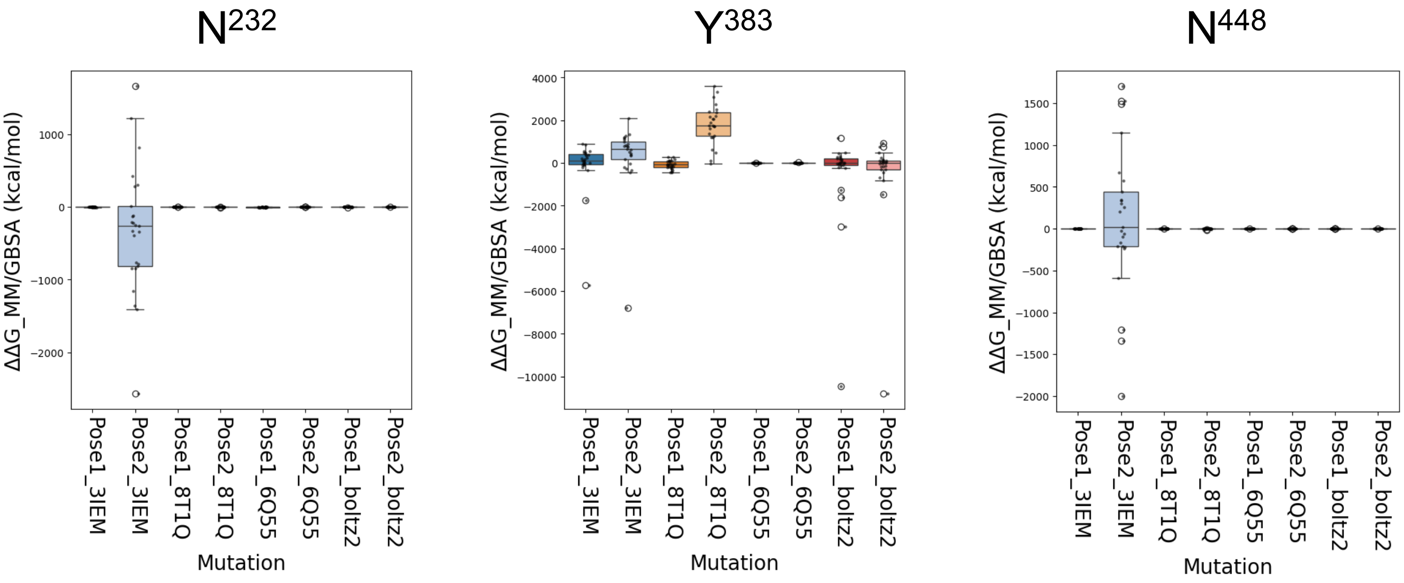

Supplementary Fig. 2: Computational modelling of acoziborole ligand affinity following mutation at the sites indicated in the CPSF3 homology models. Pose 2, for the 3IEM-based model has the greatest impact following mutations at these sites.
