# Supplementary material for: Acoziborole resistance associated mutations in Trypanosoma brucei CPSF3
Source: PLoS Pathog. 2026 Mar 3;22(3):e1013764. doi: 10.1371/journal.ppat.1013764 (PMC12970967; doi:10.1371/journal.ppat.1013764)
Supplement: S1 Table — The set of twenty-six single-stranded oligodeoxynucleotides used for oligo targeting are shown. Also shown are the primers used to generate the CPSF3 amplicon, and the primer used for Sanger sequencing. (PDF) [file ppat.1013764.s003.pdf]

Name	Sequence
CPSF3_ $V^{50}X$	AGCGGACAACCACACACGAACGGCC**NNN**TTCCCCGCCGCTGCCGATCGGTAGA
CPSF3_ $P^{72}X$	GAAGAGAATCTAATCCGCTCTTCGC**NNN**GTGGTTCCCGCAGTCCAGCATAACG
CPSF3_ $H^{100}X$	ACGGCAGTGCTCCACAGTGATCGAG**NNN**AAAGTGAGTGATGAGCACAAGGTCA
CPSF3_ $L^{101}X$	AGTACGGCAGTGCTCCACAGTGATC**NNN**GTGAAAGTGAGTGATGAGCACAAGG
CPSF3_ $D^{102}X$	AAAAGTACGGCAGTGCTCCACAGTG**NNN**GAGGTGAAAGTGAGTGATGAGCACA
CPSF3_ $H^{103}X$	CGCAAAAGTACGGCAGTGCTCCACA**NNN**ATCGAGGTGAAAGTGAGTGATGAGC
CPSF3_ $H^{183}X$	CCATGAAAAGTGCAGCCCCAAGAAC**NNN**ACCGGCATTGAAAGGCTGGAAATGA
CPSF3_ $D^{204}X$	GGTGACGGTCGGGAACACGTGAGAA**NNN**ACCTGTATATAATAGTTTCATCCCC
CPSF3_ $T^{232}X$	GTGACTCCAGTTCGCGTATACCATT**NNN**ACTCTCTGCAATGAGAATATCGGGC
CPSF3_ $N^{232}X$	CACGTGACTCCAGTTCGCGTATACC**NNN**TGTACTCTCTGCAATGAGAATATCG
CPSF3_ $R^{235}X$	CCCGCTCTTCACGTGACTCCAGTTC**NNN**TATACCATTTGTACTCTCTGCAATG
CPSF3_ $L^{237}X$	GGGATTCCCGCTCTTCACGTGACTC**NNN**TTCGCGTATACCATTTGTACTCTCT
CPSF3_ $F^{266}X$	GAAGTTCTTGCGCGCGGCCCAGAGC**NNN**TACTGGTACCAAACATCGTCCACCC
CPSF3_ $L^{268}X$	TGAGAAGAAGTTCTTGCGCGCGGCC**NNN**AGCAAATACTGGTACCAAACATCGT
CPSF3_ $R^{270}X$	CAAGTATGAGAAGAAGTTCTTGCGC**NNN**GCCCAGAGCAAATACTGGTACCAAA
CPSF3_ $M^{357}X$	GTTCAAGTGATATACCCGACTGTAG**NNN**ACCAGGAGAAGCCAACACAACGCAT
CPSF3_ $Y^{383}X$	CCTTGGCGATGGTCCCGTCAACGCA**NNN**GCCAGCAACAATAATGCCATTCCGT
CPSF3_ $V^{385}X$	GAATGTCCTTGGCGATGGTCCCGTC**NNN**GCAATAGCCAGCAACAATAATGCCA
CPSF3_ $V^{418}X$	TGCCATCGGAGTGAGCGGAGAAGGA**NNN**GGATTGAATTGTTCTCATACGGAGA
CPSF3_ $F^{420}X$	TCTGTCTGCCATCGGAGTGAGCGGA**NNN**GGAGACGGATTGAATTGTTCTCATA
CPSF3_ $S^{421}X$	GTGTCTGTCTGCCATCGGAGTGAGC**NNN**GAAGGAGACGGATTGAATTGTTCTC
CPSF3_ $A^{422}X$	CGCGTGTCTGTCTGCCATCGGAGTG**NNN**GGAGAAGGAGACGGATTGAATTGTT
CPSF3_ $H^{423}X$	AGTCGCGTGTCTGTCTGCCATCGGA**NNN**AGCGGAGAAGGAGACGGATTGAATT
CPSF3_ $H^{446}X$	GTTGCCCCATTGCCCCAACGTTGCC**NNN**TACAAGAATGACATGTTTTGTCTTG
CPSF3_ $G^{447}X$	TCAGTTGCCCCATTGCCCCAACGTT**NNN**ATGTACAAGAATGACATGTTTTGTC
CPSF3_ $N^{448}X$	TTTTCAGTTGCCCCATTGCCCCAAC**NNN**GCCATGTACAAGAATGACATGTTTT
CPSF3_$N^{232}H$	CACGTGACTCCAGTTCGCGTATACC**ATG**TGTACTCTCTGCAATGAGAATATCG
CPSF3_$R^{235}H$	CCCGCTCTTCACGTGACTCCAGTTC**ATG**TATACCATTTGTACTCTCTGCAATG
CPSF3_$L^{237}H$	GGGATTCCCGCTCTTCACGTGACTC**ATG**TTCGCGTATACCATTTGTACTCTCT
CPSF3_$V^{418}I$	TGCCATCGGAGTGAGCGGAGAAGGA**AAT**GGATTGAATTGTTCTCATACGGAGA
CPSF3_$N^{448}E$	TTTTCAGTTGCCCCATTGCCCCAAC**TTC**GCCATGTACAAGAATGACATGTTTT
CPSF3_$N^{232}Y$	CACGTGACTCCAGTTCGCGTATACC**ATA**TGTACTCTCTGCAATGAGAATATCG
CPSF3ampF	AGTAACGCTTAGCTTCTGAG
CPSF3ampR	TCAGGCAACCATCAGATGTC
CPSF3 mid seq	GTATACGAATGGCTTCTTGG

Supplementary Table 1: Oligonucleotides used in this study. The set of twenty-six single-stranded oligodeoxynucleotides used for oligo targeting are shown. Also shown are the primers used to generate the CPSF3 amplicon, and the primer used for Sanger sequencing.
