# Supplementary material for: Acoziborole resistance associated mutations in Trypanosoma brucei CPSF3
Source: PLoS Pathog. 2026 Mar 3;22(3):e1013764. doi: 10.1371/journal.ppat.1013764 (PMC12970967; doi:10.1371/journal.ppat.1013764)
Supplement: Attachment — Submitted filename: Reviewer response 02987.pdf [file ppat.1013764.s005.pdf]

PPATHOGENS-D-25-02987
Acoziborole resistance associated mutations in *Trypanosoma brucei* CPSF3

Reviewers' Comments:
Part I – Summary

Reviewer 1:
This is a very clean study identifying mutations that could impact the activity of acoziborole in T. brucei. The work is technically well-performed and the manuscript well-structured and written. Overall, it is very nice, although the results are not particularly remarkable (but few really are).

Part II – Major Issues: Key Experiments Required for Acceptance

Reviewer 1: none

Part III – Minor Issues: Editorial and Data Presentation Modifications

Reviewer 1:
With the exception of 1 and 2, these are just suggestions with respect to emphasis - things the authors might consider

1.1: The titles refer to "trypanosome" CPSF3 when only the T. brucei CPSF3 was studied. The results may not apply to other trypanosomes
R1.1: Fair point. We've adjusted the title to "…*Trypanosoma brucei* CPSF3".

1.2: The authors indicate that the approach to making triple mutants was "novel" in several places - which strictly speaking might be true but is perhaps a bit of an oversell. It was an obvious approach. By this criterion, every new experiment/approach that had not been explicitly performed previously is "novel"
R1.2: Good point. Three instances of 'novel' have been removed.

1.3: a little more discussion of why they think the S412 mutant did not confer any detectable resistance might be useful.
R1.3: We've added the following further discussion on S^{421} and N^{448} and added two additional panels to Figure 4 (D): "Our predictions also potentially explain why we failed to recover mutations at S^{421} above; because the side chain is not predicted to directly face the ligand (Fig. 4D, upper panel). We can also see why N448 does not appear in the molecular dynamics interaction network (Fig. 4A). Although N^{448} appears in close proximity to the ligand in the initial structural model, this proximity is predicted to be transient due to a highly flexible and dynamic loop (Fig. 4D, lower panel). While mutation at N^{448} alone is predicted to have no impact on RMSD (Fig. 4B) or free energy (Fig. 4C), mutation at N^{448} is predicted to impact free energy in a synergistic manner with N^{232}H and Y^{383}F (Fig. 4C), suggesting that these additional mutations impact the flexible loop. This is also consistent with recovery of N^{448} mutations only in the context of triple mutants above (Fig. 2A)".

1.4: While the authors don't hide the fact, they also don't emphasize that making the triple mutant was only possible because single mutations in these sites had been discovered in other organisms - but where not discovered using their approach in T. brucei. That is, without these other mutations to test, this paper largely wouldn't exist (the N232 mutant was reported in the Altmann 2022 paper). This represents a potential limitation of this experimental approach (it is rare to have other drug resistance info from so many other organisms in response to the same class of drugs).
R1.4: We agree to some extent but we did discover distinct mutations here. We started with a homology model that "revealed twenty-six amino acids located within 5 Å of the acoziborole

binding site in [*T. brucei*] CPSF3". We then say that the "mutations in Apicomplexan CPSF3 … are equivalent to sites targeted for editing above in *T. brucei*; N^{232}, Y^{383}, S^{421}, and N^{448}". The combinatorial oligo targeting approach could have been used with the full set of degenerate ssODNs (or a subset) without this prior knowledge, but we can only speculate as to whether that approach would have yielded informative outputs. To address this point, we've added the following text:
"Combinatorial oligo targeting proved to be effective for editing *T. brucei CPSF3* and could be used to assess other drug targets in trypanosomatids. Although insights from Apicomplexan parasites guided our analysis here in terms of targeting Y^{383} and N^{448}, the mutations we observe at these sites are distinct from those drug-resistance associated mutations previously observed in Apicomplexans; $Y^{383}F$ in *T. brucei* as opposed to $Y^{483}N$ in *Toxoplasma* and $Y^{408}S$ in *Plasmodium*, and $N^{448}H$ or $N^{448}Q$ in *T. brucei* as opposed to $E^{545}K$ in *Toxoplasma* and $D^{470}N$ in *Plasmodium*. This was possible because we used degenerate ssODNs to target Y^{383} and N^{448} for site saturation mutagenesis in *T. brucei*. Thus, known drug-resistance associated mutations in Apicomplexans guided this study, but pools of degenerate ssODNs used in combinatorial oligo targeting format could be informative even without such prior knowledge".

1.5: The difficulty in inducing and selecting for resistance to this class of compounds in trypanosomes is pretty remarkable. The authors note this, but this point feels quite undersold - and I think more comment in this regard would emphasize better the impact of this study (which otherwise is modest).
R1.5: We agree that this is remarkable and suggest that "acoziborole could be a durable monotherapy" (see the second paragraph in the discussion beginning "Our gene editing screen using oligo targeting…"). We've added some additional points here to emphasise; "which contrasts to forty-six distinct drug-resistance associated mutations identified recently in the *T. brucei* proteasome β5 subunit using a similar approach", "perhaps because the drug-binding pocket in CPSF3 is highly constrained to maintain RNA-binding" and "while the $N^{448}Q$ mutation requires a double-nucleotide change".
